# Perception of primary health professionals about Female Genital Mutilation: from healthcare to intercultural competence

**DOI:** 10.1186/1472-6963-9-11

**Published:** 2009-01-15

**Authors:** Adriana Kaplan-Marcusan, Pere Torán-Monserrat, Juana Moreno-Navarro, Ma Jose Castany Fàbregas, Laura Muñoz-Ortiz

**Affiliations:** 1 Department of Social and Cultural Anthropology. Autonomous University of Barcelona, 08193 Bellaterra, Barcelona, Spain; 2 Primary Healthcare Centre Gatassa (Mataró-6), Catalan Health Institute, Camí del Mig 36 (4^a ^planta), 08303 Mataró Barcelona, Spain; 3 Primary Healthcare Centre Sant Andreu de Llavaneres. Catalan Health Institute, Passeig Jaume Brutau s/n, 08392, Sant Andreu de Llavaneres Barcelona, Spain; 4 Primary Healthcare Research Support Unit of Barcelonès Nord i Maresme. IDIAP Jordi Gol, Camí del Mig 36 (3^a ^planta), 08303 Mataró Barcelona, Spain

## Abstract

**Background:**

The practice of Female Genital Mutilation (FGM), a deeply-rooted tradition in 28 countries in Sub-Saharan Africa, carries important negative consequences for the health and quality of life of women and children. Migratory movements have brought this harmful traditional practice to our medical offices, with the subsequent conflicts related to how to approach this healthcare problem, involving not only a purely healthcare-related event but also questions of an ethical, cultural identity and human rights nature.

**Methods:**

The aim of this study was to analyse the perceptions, degree of knowledge, attitudes and practices of the primary healthcare professionals in relation to FGM. A transversal, descriptive study was performed with a self-administered questionnaire to family physicians, paediatricians, nurses, midwives and gynaecologists. Trends towards changes in the two periods studied (2001 and 2004) were analysed.

**Results:**

A total of 225 (80%) professionals answered the questionnaire in 2001 and 184 (62%) in 2004. Sixteen percent declared detection of some case in 2004, rising three-fold from the number reported in 2001. Eighteen percent stated that they had no interest in FGM. Less than 40% correctly identified the typology, while less than 30% knew the countries in which the practice is carried out and 82% normally attended patients from these countries.

**Conclusion:**

Female genital mutilations are present in primary healthcare medical offices with paediatricians and gynaecologists having the closest contact with the problem. Preventive measures should be designed as should sensitization to promote stands against these practices.

## Background

The World Health Organization (WHO) has calculated that 100 to 140 million women and girls throughout the world have been victims of some form of Female Genital Mutilation (FGM) in an extensive area of Sub-Saharan Africa and in some Asian countries (Indonesia, Malaysia) and the Middle East (Yemen, Arab Emirates, Iraq). Each year around 3 million girls are at risk or are submitted to some type of excision, mainly in the 28 countries in Sub-Saharan Africa in which these ancestral rituals are deeply-rooted [1,2].

According to a report by the Innocenti Research Centre [3], FGM continues to be one of most persistent and omnipresent violations of human rights, which is silently tolerated and has an important impact on the health of the women affected [4]. FGM is, therefore, a healthcare problem which surpasses the purely healthcare framework since it includes the infringement of human rights and the need for a transcultural approach to questions closely linked to ethnic identity and gender.

The substantial migratory flow of the Sub-Saharan population towards Europe in recent years is leading to increasingly more complex and diverse societies. The approach to the healthcare problems affecting this population represents a challenge to healthcare systems [5-7] and the professionals working therein, who must develop their own competence to achieve transcultural care [8-10].

It is not continents or colours that emigrate but rather people and their cultures, in recent years a visible distortion has been produced in the phenomena associated with gender and immigration, especially in the case of Sub-Saharan women, their daughters and the harmful traditional practices of initiation involving mutilation of female genitals. International organisations and professional associations have made statements against FGM [1,11]. Indeed, many countries have promulgated legislation against these practices. In Spain the law punishes this crime with prison sentences of 6 to 12 years for the parents, and the girls are taken into care by Social Services.

In addition to adequate laws, a preventive stand is essential which, from a perspective of knowledge and sensitisation, allows healthcare professionals to approach the question of FGM and thereby avoid the conflicts produced by legal action against aspects such as those linked to the intimacy and identity of these people.

The first cases of FGM in Spain were detected and reported by healthcare professionals in 1993. Since then, new mutilations have not been reported in Spain, although it is known that some families take advantage of vacation trips to their countries of origin to carry out FGM. From the national census, from countries in which these practices are carried out, residing in Spain in 2005, we have estimated that in our country around 9,545 women have undergone some type of FGM and approximately 3,824 girls are at an age of risk of having this done within the next few years. Not taking into account the rise in the population due to the latest legislation related to the regularization of immigrants.

This emerging sociodemographic reality has led to new challenges for which healthcare professionals should be prepared to intervene from a preventive point of view. The FGM are a problem which affects us, as healthcare professionals, in a double sense, as a violation of human rights, which we have the moral obligation to impede, and as an aggression against the health of the persons to which we have the professional obligation to attempt to prevent, to thereby avoid the consequences to the physical, psychic, sexual and reproductive well being of the women [4,11-16].

The cultural pressure and social structure which these practices maintain are strong since they are rooted and nourished by the tradition and the previous experience of their elders, their mothers and confusing religious messages in their communities of origin.

We believe that the receptor countries should approach the question of the FGM from any of the possible points of contact of the migrant families with Primary Health Care, advancing in the double line of:

• Training the professionals in detection and recognition and preventive intervention in the families and girls in a situation of risk of undergoing FGM.

• Identify the women and girls at risk in the population assigned to each healthcare centre.

Because of its proximity to the families and the longitudinal approach to the problems throughout the whole vital cycle, primary healthcare is one entry point for implementing a preventive intervention towards FGM [17].

The objectives of this study were to analyse the perceptions, degree of knowledge and attitudes of primary care professionals related to FGM, as well as explore possible trends towards a change in these perceptions and attitudes in two periods of time, 2001 and 2004.

## Methods

A cross-sectional study was conducted with a self-administered questionnaire to primary healthcare professionals. In the Catalonian Healthcare System, primary healthcare professionals work in teams who attend the healthcare needs of the population assigned in a determined territory. These teams are made up of family physicians/general medicine, paediatricians, nurses and social workers. Support programs are available within the setting of sexual and reproductive health and these are mainly constituted by gynaecologists and midwives. We considered three large groups of professionals according to the characteristics of the population they attend: General Medicine (physicians and nurses) attending a population over the age of 15 years; Paediatrics (physicians and nurses) attending a population under the age of 15 years and Gynaecology (gynaecologists, nurses and midwives) attending the program of sexual and reproductive health.

Two time points were used: April-May 2001 and October-November 2004. Between the two time points the results of the first questionnaire were made public, and some educational activities were carried out (seminars, sessions and courses) in the healthcare centres, although these activities were not part of a structured training programme related to FGM. In June 2002 a "Protocol of action to prevent FGM" was edited on behalf of the autonomous Government of Catalonia [18] and in January 2004 guidelines for professionals were presented within the framework of a European project against sexual violence [19].

The study was undertaken in the Maresme, a county on the Mediterranean coast north of Barcelona with a population of 412,840 inhabitants. This area was one of the first areas to receive large groups of Sub-Saharan immigrants, mainly from Gambia and Senegal during the migratory wave produced at the beginning of the 1980s. According to the census of 2005, 11.28% of the population of the county was foreigners, 30.24% of whom were from North Africa and 13.44% from Sub-Saharan Africa.

The professionals surveyed belonged to the departments of General Medicine, Paediatrics, Gynaecology, and Social Work in 13 healthcare centres of the public network of the Catalonian Institute of Health. All healthcare professionals (physicians, paediatricians, nursing staff, midwives and social workers) from 13 primary healthcare teams and the Programme of Healthcare for Sexual and Reproductive Care were included in the study.

The strategy of questionnaire distribution consisted in contacting representatives in each centre, who received the questionnaires and were in charge of distribution among all the components of the respective teams. A member of the research team previously contacted these people by telephone to explain the objectives of the study. Two weeks later a telephone reminder was made and collection of the completed questionnaires was arranged. A total of 280 questionnaires were distributed in 2001 and 296 in 2004, corresponding to all staff members of the participating centres.

The questionnaire (Additional file 1) collected information on sociodemographic variables (age, gender, profession and speciality), degree of knowledge on FGM (identification and typology, reasons for this practice, countries in which it is carried out), degree of interest elicited (need or desire to know more on the subject, performance of educational activities or knowledge of protocols of guidelines of action), previous experience (care to patients from countries in which FGM is performed, detection of any case) and attitudes versus FGM (ignore, educate-sensitise, report to authorities). The 2004 edition did not include the questions exploring the knowledge of the reasons for which FGM is carried out and the countries in which it is performed (questions 3–5), since it was considered that sufficient information was obtained with the questions of global knowledge and identification of the types of FGM (questions 1 and 2).

### Statistical methods

The questionnaires were anonymous and the participants in the two-year time points could not be identified. We performed all the comparisons using robust standard errors considering the 3 professionals groups (General Medicine, Paediatrics and Gynaecology) as clusters. This statistical analysis allows the independence assumption about the data to be relaxed and requires only that the observations are independent across the clusters. First a descriptive analysis was carried out, stratifying the variable studied by year. Secondly, to identify the changes in knowledge, attitude and interest of the professionals, the total proportions of these variables were compared between the two years, also stratified by gender, age, group and speciality of the professionals with the Chi-square test. To identify the characteristics of the professionals which may influence correct identification (CI) of the FGM, the detection of cases (DC) and the attitude (AT) of these professionals, a multiple logistic regression model, based on robust standard errors considering the 3 professionals groups as clusters was used with CI, DC or AT as dependent variables and gender, age, professional group, attendance to a Sub-Saharan population, belief that this was performed for traditional or religious reasons and if interested and performed educative activities on FGM as independent variables. A different model was adjusted for each dependent variable. Statistical significance was considered at p < 0.05, based on robust standard errors. The analyses were performed using Stata software, version 9.2 (StataCorp, College Station, TX, USA).

### Ethics

We carried out the survey in professionals who were orally informed in their centres as to the objectives and the content of the questionnaire prior to doing it. Participation was voluntary. The information was treated totally anonymously so that it was impossible to identify the identity of the participating professional through the answers provided. Neither were the professionals who had decided not to participate identifiable. There was no direct intervention (pharmacologic, educational, formational or preventive) in patients or users of the healthcare system or in professionals except for knowing their opinions. No external financial support was required. The management of the centres were informed of the study and the manuscript was sent to the Ethics Committee of our institution.

## Results

Response was obtained in 80% and 62% of the questionnaires distributed in 2001 and 2004, respectively. The sociodemographic and professional characteristics of the responders as well as the knowledge, attitudes and interest related to FGM stratified by gender are shown in Tables 1 and 2. No statistically significant differences were found between the participants in the two years except for the 20–40 and 41–50 age groups, number of professionals with cases detected and in the attitude of the professionals with respect to the FGM. It was observed that 90.1% normally attended a population from Morocco and 79.8% patients of Sub-Saharan origin.

**Table 1:** Sociodemographic and professional characteristics, knowledge, attitudes and interest related to FGM among the study participants.

	**2001**		**2004**	
	
	**N**	**%**	**N**	**%**
**Distributed questionnaires**	280		296	
**Returned questionnaires**	225	80%	184	62%
**Gender**				
Males	63	28.0	50	27.2
Females	155	68.9	131	71.2
Unknown	7	3.1	3	1.6
**Age **(years)				
20 – 40	54	24.0	58	31.5*
41 – 50	121	53.8	80	43.5*
> 50	44	19.5	42	22.8
Unknown	6	2.7	4	2.2
**Professional group**				
General medicine	162	72.0	140	79.6
Paediatrics	42	18.7	27	15.3
Gynaecology	18	8.0	5	2.8*
Unknown	3	1.3	4	2.3
**Speciality**				
General or Family Medicine	77	34.2	70	38.0
Gynaecology	7	3.1	4	2.2
General Nursing	85	37.8	70	38.0
Paediatric Nursing	22	9.8	15	8.2
Paediatrician	20	8.9	12	6.5
Midwife	11	4.9	1	0.5
Social worker	-	-	8	4.4
Unknown	3	1.3	4	2.2
**Correctly identified**				
FGM typology	100	45.2	70	40.0
Countries of FGM	42	23.9		
**Have detected some case**	13	5.9	30	16.3*
**Attitudes**				
Ignore	2	0.9	0	0,0
Educate	112	49.8	57	31.0*
Report to authorities	40	17.8	44	23.9*
Educate and report	71	31.5	83	45.1*
**Interest**	173	82.4	115	82.7

Percentages calculated from questionnaires with information.

* p < 0.05 between 2001 and 2004

**Table 2:** Knowledge, attitudes and interest related to FGM according to gender.

	**2001**		**2004**		**Total**		
		
	**Males**	**Females**	**Males**	**Females**	**Males**	**Females**	**p**^1^
		
	%	%	%	%	%	%	
**KNOWLEDGE**							
							
State they know what FGM is	98.4	98.1	98.0	94.7*	98.2	96.5	< 0.001
Correctly identify	35.5	48.7	40.8	39.5*	37.8	44.6	0.001
							
State they know the countries	80.9	77.8	-	-	-	-	0.579
Correctly identify	17.6	26.9					0.188
							
Believe FGM performed for:							
Religious reasons	19.7	13.2					
Tradition	45.9	54.6					
Religious reasons and Hygiene	1.6	-	-	-	-	-	0.317
Religious reasons and Tradition	29.6	27.0					
Hygiene and Tradition	1.6	3.9					
All	1.6	1.3					
							
Attend population from:							
Morocco	90.0	89.7	-	-	-	-	0.661
Sub-Saharan Africa	86.8	77.1	-	-	-	-	< 0.001
Have detected some case	3.2	6.6	6.0	20.6*	4.4	13.1	0.009
							
**ATTITUDES**							
							
Ignore	1.6	0.6	0.0	0.0	0.9	0.3	0.004
Educate	44.4	51.6	18.0*	36.6*	32.7	44.8	
Report to authorities	22.2	15.5	22.0	25.2	22.1	19.9	
Educate and report	31.8	32.3	60.0*	38.2*	44.3	35.0	
							
**INTEREST**	69.5	87.0	81.1*	83.2	74.0	85.4	0.001
**FORMATION**							
							
Have received training	-	-	10.0	15.3	-	-	< 0.001
Know some protocol of action	-	-	22.5	18.4	-	-	0.554

^1 ^p between males and females.

* p < 0.05 between 2001 and 2004

Almost 18% of those surveyed expressed a lack of interest in the subject, with a significantly greater interest demonstrated by females (p = 0.001). Ninety-eight percent of the males and 96% of the females claimed to know what FGM was but only 40% correctly identified the types of FGM and less than 30% could identify the countries in which this practice was performed. A statistically significant increase was observed among the professionals who detected cases in 2001 (5.9%) and in 2004 (16.3%), with women being those who referred the greatest rates of detection of FGM in both years (p = 0.009).

A statistically significant decrease was observed in the grade of knowledge of FGM (98.1% in 2001, 94.7% in 2004) and in relation to the correct identification of the typology of FGM (48.7% in 2001, 39.5% in 2004) among the women.

Of those surveyed in 2001 and 2004, 49.8% and 31% respectively, believed that the attitude of the healthcare professionals towards FGM should be educational and that patients should be sensitised, 17.8% and 22.9% reported cases to the authorities and 31.5% and 45.1% stated that the two strategies should be combined. On comparing both years the differences were statistically significant (p < 0.001).

According to the professional group, 38.3% of the staff of general medicine, 55.1% of paediatrics and 68.2% of those in gynaecology correctly identified the typology of FGM. Three point seven percent of the professionals from general medicine, 7.3% of those from paediatrics and 22.2% of those from gynaecology declared the detection of girls or women with problems related to FGM in 2001; with these percentages rising to 12.1%, 18.5% and 80%, respectively in 2004. Personnel in the field of paediatrics demonstrated the greatest interest (91%) and better knowledge of the guidelines and protocols of action (42.3%).

Additional file 2 shows the knowledge, attitudes and practices related to FGM according to professional group and speciality. Nurses identified the different types of FGM better than the physicians in all the professional groups in both years, except for gynaecology in 2001.

The characteristics of the professionals who may have influenced in the correct identification of FGM, the detection of cases and attitudes are shown in Additional file 3. The professionals who had attended some educative activity on FGM or were familiar with any protocol or guideline of action had up to a 5.0-fold greater probability to correctly identify the typology.

The detection of cases was mainly undertaken by female professionals (OR = 1.9; CI 95% [0.8–4.4]) of less than 40 years in age. Females identified the FGM better than males (OR = 1.6; CI 95% [1.5–1.7]), developed educational attitudes from the approach to the FGM (OR = 1.8 CI 95% [1.4–2.2]), and declared greater interest in the subject of FGM (OR = 2.1; CI 95% [1.6–2.8]).

The gynaecologists demonstrated better knowledge of the FGM (OR = 2.8; CI 95% [2.7–2.9] and a greater probability of case detection (OR = 8.5; CI 95% [4.9–14.7].

## Discussion

This study demonstrates that the problems related to FGM are not infrequent in primary care consultations since up to 16% of the participants surveyed in 2004 declared having detected cases. Eighteen percent of the professionals declared no interest in knowing more about the subject. Less than 40% correctly identified the typology and less than 30% knew the countries in which this practice was common. There seemed to be greater sensitivity towards the subject by female nurses and midwives within the professional areas of paediatrics and gynaecology. A trend towards education and sensitisation versus the problem was observed accompanied by reporting to the authorities when these preventive approaches failed.

The percentage of professionals who declared having detected a case practically tripled from 2001 to 2004, although these values are far from the 60% declared in a questionnaire to professionals from four Swedish cities [20].

It should be taken into account that our questionnaire only allowed exploration of whether had diagnosed or had knowledge of any child in their office that had undergone FGM. We could not determine the number of cases diagnosed. We only considered the number of professionals who, in their practice and based on their clinical practice, had diagnosed or known of any child with FGM.

In the Swedish study [20] a very low rate of response was obtained (28%) and it cannot be ruled out that the professionals who responded had been in contact with cases of FGM and were especially motivated or sensitised with the subject. In our study the rate of response was much higher, which we believe better represents the opinion of most of the professional groups. Moreover, our study population included a lower proportion of gynaecologists and midwives than the Swedish study, which may explain the lower percent of professionals in contact with cases. Knowledge of FGM seemed to be similar in the two populations. In the Swedish survey 35% of the gynaecologists and 29% of the midwives declared having "sufficient knowledge" of the subject. In our study 45% of the participants correctly identified the typology and 24% the countries of origin. The difference lays in that in our study the data was obtained from the emission of correct responses, similar to an examination, to two questions related to this subject, while the Swedish questionnaire concerned the perception of "sufficient knowledge" on behalf of the professionals.

In a study carried out in Switzerland [6] only 8% of the 37 professionals interviewed referred having developed any preventive intervention in their consultations. In our questionnaire in 2004, 31% of the professionals expressed the need to develop educational and sensitisation attitudes to avoid FGM. We believe that these differences may be due to the limited sample size, being mainly of professionals specialised in gynaecology in the Swiss study, while in our study the collective analysed included primary care professionals with a more general view of healthcare problems, being theoretically closer to preventive and educational healthcare activities.

The discrepancy between the perception of having correct knowledge (knowing in which countries FGM is performed and for what reasons) and the correct identification of the typology, the countries and the reasons for its performance, should be pointed out. This indicates that, in fact, the professionals in our environment have a significant lack of knowledge with regard to FGM. If we add the fact that 80% of those surveyed stated that they attended a population of Sub-Saharan origin and that the detection of cases tripled from 2001 to 2004, it may be deduced that we are faced with an emerging healthcare problem which we are treating with a great lack of knowledge on its social and cultural background.

Our results demonstrate that the primary care professionals surveyed declare a high interest (more than 70% answered "yes" to the question as to whether they are interested in knowing more on this subject), a low grade of knowledge (less than half correctly identified the types of FGM and less than 25% correctly identified the countries in which it is practiced) and some efficacy in the formation received (those stating that had received formation had a greater probability of better identifying the typology and origin, according to model of logistic regression in Additional file 3). This leads to the need to prioritize strategies of sensitization and formation of professionals in relation to the identification of the population at risk and capacitation for a preventive and culturally respectful approach, to thereby avoid the families being exposed to the criminal and legal procedures linked to their socio-cultural roots and the consequent risk of familial destruction and separation.

In addition, in our country it so happens that the fertility of African women of Gambian origin doubles or triples that of women from other areas [21]. To develop effective interventions in the immediate future it will be necessary to promote in depth knowledge of the social and cultural reality of these migrant communities [10,22] which will allow these subjects to be approached with greater professional competence and thus, greater possibilities of success.

One interesting fact is the difference observed in the perception, attitudes and detection of the problem according to the gender of the professionals. The women showed greater interest, had more attitudes oriented towards education and detected most of the cases. These differences were maintained after adjustment with multivariate analysis. In the case of the women a certain decline was observed in the grade of knowledge and interest related to FGM. Although both values were high (around 84% interest and 95% knowledge), the trend was to the contrary in the case of the men, with a clear trend to an increase being found. The change towards to educational attitudes was consistent in both the men and women. We believe that this may reflect a certain sensitization of the professionals to this problem, following a more accelerated dynamics in the case of the women (possibly for questions of gender solidarity) than in the case of the men, who are still in a process of awareness related to the problem.

The different perspective according to the gender of the professionals should be taken into account to avoid an increase in certain disparities in healthcare based on the professionals attending the migrant women population. The challenge lays in avoiding the creation of another barrier in the approach to problems related to FGM due to this difference in gender in the perception of the professionals in relation to FGM. The results of our study indicate that to avoid these inequities men must be sensitized, cultural stereotypes should be opposed and we must be respectful as to the preferences of the migrant women with regard to the gender of the professional they wish to be attended by, similar to what has been observed in other types of problems [23,24].

The fact of having received previous training or knowing of protocols of action seems to be, as expected, associated with a greater identification of the types of FGM but not with a greater detection of cases. In the professionals with previous training, attitudes toward reporting to authorities rather than education were also observed. Although these differences were not statistically significant, we believe that they mark a trend and may represent a certain degree of theoretical knowledge, albeit a lack of practical skills toward the approach, detection and prevention.

Catalonia has been a receptor of Sub-Saharan immigration since the 1980s and at the end of this decade the first phenomena of family regrouping took place with the arrival of the wives and children. Legislation against FGM in Spain was promulgated in 2003 and a law allowing extraterritorial persecution of this crime came into effect in 2005. We are, therefore, before a recent and culturally alien phenomenon for which practicing physicians have not received training.

The results of this study demonstrate that the way the primary care professionals in our area confront FGM is similar to that of their colleagues in five European countries studied by Leye [7]. It also describes important gaps in knowledge and cultural contextualisation and scarce educational measures and support for decision making, with important ethical dilemmas which make not only a clinically adequate professional approach but also culturally respectful approach towards the beliefs and needs of the women.

With regard to the limitations of this study it should be indicated that the anonymity of the questionnaire does not allow paired analysis between the professionals participating in 2001 and those of 2004. It should also be mentioned that the questionnaire in 2004 did not collect exactly the same information on the knowledge, reasons and countries in which this practice is common, and therefore we could not compare this information between the two years. We believe that the lack of statistical significance in some of the trends observed on multivariate analysis was due to a lack of study power to detect these differences.

## Conclusion

This study demonstrates that the problem of FGM is present in the primary care centres in our country, with the percentage of professionals detecting some case having tripled in three years. The collectives most in contact with the problem are those in paediatrics and gynaecology, with women showing greater professional sensitivity towards the subject. The professionals over-evaluated their degree of knowledge of FGM and less than half could correctly identify the typology and countries in which FGM is practiced. It is therefore necessary to promote anthropologic knowledge of the problem to develop activities of prevention and detection of family risk to avoid legal actions against these subjects. The development of positive models of intervention, with respectful transcultural attention to values and beliefs in subjects as culturally deep-rooted and sensitive as FGM, will also allow other cultural aspects linked to health and disease among immigrants to be approached more successfully. This problem has only recently appeared in Spain and should be analysed and monitored with new studies to observe in depth the attitudes of the professionals towards this situation, and to also explore the beliefs and needs of the immigrant families in our countries.

## Keypoints

• Female genital mutilation (FGM) and its consequences in the health of women are emerging healthcare problems that are present in primary care centres.

• Healthcare professionals approach this question with a profound lack of knowledge as to the social and cultural foundations of FGM.

• Migratory movements and demographic trends of the migrant populations will, in the not too distant future, lead to many healthcare professionals being faced with ethical-legal dilemmas related to the approach to FGM.

• The development of transcultural healthcare models will facilitate a preventive approach to avoid legal recourse of these cases and the negative consequences of family destabilization.

• Female professionals present greater rates of detection of FGM and a greater sensitivity in their approach to this problem.

## Competing interests

The authors declare that they have no competing interests.

## Authors' contributions

AKM is a principal investigator of the GIPE/PTP work group; AKM, JMN, MJCF participated in the design of the study, in the field work for acquisition of data and in the results interpretation; LMO performed the statistical analysis and methodological support to study design; PTM helped analyzed data, participated in the results interpretation and drafted the manuscript. All the authors have read, revised and approved the final manuscript and have agreed to its submission for publication.

## Pre-publication history

The pre-publication history for this paper can be accessed here:



## Supplementary Material

Additional File 1 **Annex 1**. Questionnaire. Questionnaire used in the survey. Click here for file

Additional File 2 **Table 3**. Knowledge, attitudes and interest related to FGM according to professional group and speciality. Click here for file

Additional File 3 **Table 4**. Multiple logistic regression models on knowledge, attitudes and interest related to FGM and different independent variables (2001–2004). Click here for file

## References

[B1] Inter-Agency (2008). Eliminating Female Genital Mutilation An Interagency Statement.

[B2] World Health Organization (1997). Female Genital Mutilation.

[B3] United Nations Children's Found (2005). Changing a harmful social convention: Female Genital Mutilation/Cutting.

[B4] Obermeyer CM (2005). The consequences of female circumcision for health and sexuality: an update on the evidence. Cult Health Sex.

[B5] Löfvander M, Dyhr L (2002). Transcultural general practice in Scandinavia. Scand J Prim Health Care.

[B6] Thierfelder C, Tanner M, Bodiang CM (2005). Female genital mutilation in the context of migration: experience of African women with the Swiss health care system. Eur J Public Health.

[B7] Leye E, Powell RA, Nienhuis G, Claeys P, Temmerman M (2006). Health Care in Europe for women with genital mutilation. Health Care Women Int.

[B8] Juckett G (2005). Cross-cultural Medicine. Am Fam Physician.

[B9] Kripalani S, Bussey-Jones J, Katz MG, Genao I (2006). A prescription for cultural competence in medical education. J Gen Intern Med.

[B10] Geiger HJ (2001). Racial stereotyping and medicine: the need for cultural competence. CMAJ.

[B11] (1998). Female genital mutilation: American Academy of Pediatrics. Committee on Bioethics. Pediatrics.

[B12] Craft N (1997). Life span: conception to adolescence. BMJ.

[B13] Morison L, Scherf C, Ekpo G, Paine K, West B, Coleman R, Walraven G (2001). The long-term reproductive health consequences of female genital cutting in rural Gambia: a community-based survey. Trop Med Int Health.

[B14] el-Defrawi MH, Lotfy G, Dandash KF, Refaat AH, Eyada M (2001). Female genital mutilation and its psychosexual Impact. J Sex Marital Ther.

[B15] Dandash KF, Refaat AH, Eyada M (2001). Female genital mutilation: a descriptive Study. J Sex Marital Ther.

[B16] Walraven G, Scherf C, West B, Ekpo G, Paine K, Coleman R, Bailey R, Morison L (2001). The burden of reproductive-organ disease in rural women in The Gambia, West Africa. Lancet.

[B17] Kaplan Marcusan A, Torán Monserrat P, Bedoya Muriel MH, Bermúdez Anderson K, Moreno Navarro J, Bolibar Rivas B (2006). [Genital mutilation of women: reflections for a primary care intervention]. Aten Primaria.

[B18] Generalitat de Catalunya (2002). Protocol d'actuacions per prevenir la Mutilació Genital Femenina [Protocol for Female Genital Mutilation prevention].

[B19] Kaplan A, Martínez C, editors (2004). Mutilación Genital Femenina: prevención y atención Guía para profesionales [Female Genital Mutilation: prevention and attention A guideline for professionals].

[B20] Tamaddon L, Johnsdotter S, Liljestrand J, Essén B (2006). Swedish health care providers' experience and knowledge of female genital cutting. Health Care Women Int.

[B21] Bledsoe CH, Houle R, Sow P (2007). High fertility Gambians in low fertility Spain: The dynamics of child accumulation across transnational space. Demographic Res.

[B22] Gruenbaum E (2005). Socio-cultural dynamics of female genital cutting: research findings, gaps, and directions. Cult Health Sex.

[B23] O'Mahony JM, Donnelly TT (2007). Health care providers' perspective of the gender influences on immigrant women's mental health care experiences. Issues Ment Health Nurs.

[B24] Reitmanova S, Gustafson DL (2008). "They can't understand it": maternity health and care needs of immigrant Muslim women in St. John's, Newfoundland. Matern Child Health J.

