# Supplementary material for: Perception of primary health professionals about Female Genital Mutilation: from healthcare to intercultural competence
Source: BMC Health Serv Res. 2009 Jan 15;9:11. doi: 10.1186/1472-6963-9-11 (PMC2631456; doi:10.1186/1472-6963-9-11)
Supplement: Additional File 1 — Annex 1. Questionnaire. Questionnaire used in the survey. [file 1472-6963-9-11-S1.pdf]

QUESTIONNAIRE

Survey conducted in the year ………..

The reason for this survey, anonymous and voluntary, is to know your opinion as a health professional on **Female Genital Mutilation** or **Ablation**.

Gender: ☐ Male ☐ Female

Age: …………… years old

Speciality:

☐ General or Family medicine ☐ Paediatric nursing

☐ Gynaecology ☐ Paediatrician

☐ General nursing ☐ Midwife

☐ Social worker

---

1. Do you know what ablation is? ☐ No ☐ Yes

If your answer is **Yes**, please select the correct option:

☐ Removal of the clitoris

☐ Removal of the labia majora

☐ Removal of the labia majora and minora

☐ All of the above are true

2. Are you interested in knowing more about this topic? ☐ No ☐ Yes

3. Why do you think that the ablation is performed? (*)

☐ Religious reasons

☐ Hygiene

☐ Tradition

4. Do you know in which countries it is practiced? (*) ☐ No ☐ Yes

If your answer is **Yes**, please select which of them:

☐ Morocco

☐ Senegal

☐ Gambia

☐ Mauritania

5. Do you see patients …

… from Morocco? ☐ No ☐ Yes

… from Sub-Saharan Africa? ☐ No ☐ Yes

6. Have you met any girl to whom the ablation was performed? ☐ No ☐ Yes

7. How do you think we should react to this situation?

☐ Ignore

☐ Educate

☐ Report to authorities

8. Have you received any kind of education about this topic? ☐ No ☐ Yes

If your answer is **Yes**, which kind of formation?
…………………………………………………………………

If your answer is **NO**, do you have any interest in doing so? ☐ No ☐ Yes

9. Do you have knowledge of any protocol of actuation? ☐ No ☐ Yes

If your answer is **Yes**, specify which:
……………………………………………………………………………

Thank you for your collaboration.

MJ Castany J Moreno (ABS Llavaneres)

*** These questions were not included in the 2004 edition.**
