# Supplementary material for: Perception of primary health professionals about Female Genital Mutilation: from healthcare to intercultural competence
Source: BMC Health Serv Res. 2009 Jan 15;9:11. doi: 10.1186/1472-6963-9-11 (PMC2631456; doi:10.1186/1472-6963-9-11)
Supplement: Additional File 2 — Table 3. Knowledge, attitudes and interest related to FGM according to professional group and speciality. [file 1472-6963-9-11-S2.pdf]

Table 3. Knowledge, attitudes and interest related to FGM according to professional group and speciality.

	2001						2004					
	General Medicine		Paediatrics		Gynaecology		General Medicine		Paediatrics		Gynaecology	
	P	N	P	N	P	N	P	N	P	N	P	N
	%	%	%	%	%	%	%	%	%	%	%	%
**KNOWLDEGE**												
State they know what FGM is	97.4	98.8	100.0	100.0	100.0	90.9	91.4	95.7	100.0	100.0	100.0	100.0
Correctly identify	37.3	46.4	40.0	54.5	85.7	60.0	29.7	37.3	58.3	73.3	50.0	100.0
State they know the countries	72.4	78.8	85.0	81.8	100.0	90.9	-	-	-	-	-	
Correctly identify	23.6	20.9	29.4	38.9	14.3	20.0						
Believe FGM performed for:												
Religious reasons	19.2	21.7	5.0	4.5	-	-						
Tradition	47.9	48.2	50.0	59.2	57.1	81.8						
Religious and hygiene	1.4	-	-	-	-	-	-	-	-	-	-	-
Religious and Tradition	30.1	25.3	40.0	31.8	14.3	9.1						
Hygiene and Tradition	-	4.8	5.0	4.5	14.3	-						
All	1.4	-	-	-	14.3	9.1						
Attend population from:												
Morocco	89.3	85.7	100.0	100.0	80.0	90.9	-	-	-	-	-	-
Sub-Saharan Africa	80.9	73.2	89.5	94.7	42.9	90.9	-	-	-	-	-	-
Have detected some case	2.6	4.8	15.0	0.0	28.6	18.2	14.3*	10.0	0.0	33.0	100.0*	0.0
**ATTITUDES**												
Ignore	1.3	0.0	0.0	0.0	0.0	9.1	0.0	0.0	0.0	0.0	0.0	0.0
Educate	36.4	55.3	75.0	63.6	42.9	45.4	18.6*	37.1	41.7*	53.3	50.0	0.0
Report to authorities	25.9	17.6	0.0	9.1	14.2	18.2	25.7	25.8	33.3	6.7	25.0	0.0
Educate and report	36.4	27.1	25.0	27.3	42.9	27.3	55.7*	37.1	25.0	40.0	25.0	100.0
**INTEREST**	71.0	89.0	83.3	90.5	85.7	90.9	74.0	86.4	100.0	100.0	66.7	100.0
**FORMATION**												
Have received training	-	-	-	-	-	-	11.4	12.9	8.3	20.0	0.0	0.0
Know some protocol of action	-	-	-	-	-	-	14,3	14.5	63.6	26.7	0.0	0.0

P: Physicians; N: Nurses

* $p < 0.05$ between 2001 and 2004
