# Supplementary material for: Perception of primary health professionals about Female Genital Mutilation: from healthcare to intercultural competence
Source: BMC Health Serv Res. 2009 Jan 15;9:11. doi: 10.1186/1472-6963-9-11 (PMC2631456; doi:10.1186/1472-6963-9-11)
Supplement: Additional File 3 — Table 4. Multiple logistic regression models on knowledge, attitudes and interest related to FGM and different independent variables (2001–2004). [file 1472-6963-9-11-S3.pdf]

Table 4. Multiple logistic regression models on knowledge, attitudes and interest related to FGM and different independent variables (2001-2004)

	KNOWLEDGE		ATTITUDES			INTEREST
	CI	DC	Educate	Report	Educate and Report	
**Gender**						
Males	1	1	1	1	1	1
Females	1.6 (1.5 – 1.7)*	1.9 (0.8 – 4.4)	1.8 (1.4 – 2.2)*	0.9 (0.8 – 1.1)	0.6 (0.5 – 0.8)*	2.1 (1.6 – 2.8)*
**Age** (years)						
20 – 40	1	1	1	1	1	1
41 – 50	1.1 (0.9 – 1.4)	0.5 (0.1 – 2.0)	1.1 (0.9 – 1.4)	0.8 (0.4 – 1.5)	1.1 (0.9 – 1.4)	1.0 (0.8 – 1.1)
> 50	1.8 (1.0 – 3.6)*	0.2 (0.1 – 0.8)*	0.9 (0.6 – 1.3)	1.2 (1.0 – 1.5)	1.0 (0.7 – 1.5)	1.1 (0.9 – 1.3)
**Professional group**						
General medicine	1	1	1	1	1	1
Paediatrics	1.7 (1.6 – 1.9)*	2.3 (1.8 – 2.9)*	3.2 (3.0 – 3.3)*	0.3 (0.2 – 0.3)*	0.6 (0.5 – 0.6)*	2.6 (2.5 – 2.8)*
Gynaecology	2.8 (2.7 – 2.9)*	8.5 (4.9 – 14,.7)*	1.2 (1.1 – 1.3)*	0.7 (0.7 – 0.8)*	1.0 (0.9 – 1.1)	1.9 (1.7 – 2.0)*
**Attend population from Sub-Saharan Africa** [a]	1.7 (1.4 – 1.9)*		1.0 (0.6 – 1.5)	1.2 (1.0 – 1.4)*	0.9 (0.5 – 1.4)	1.1 (0.6 – 1.8)
**Believe FGM performed for** [a]						
Tradition	1	1	1	1	1	1
Religious reasons	0.8 (0.5 – 1.3)	1.3 (0.7 – 2.5)	0.8 (0.5 – 1.3)	1.1 (0.9 – 1.4)	1.3 (0.6 – 2.9)	0.6 (0.1 – 3.0)
Tradition and Religious reasons	1.7 (1.5 – 1.9)*	1.0 (0.1 – 8.6)	0.5 (0.3 – 1.0)	1.3 (0.9 – 2.0)	1.7 (0.6 – 4.8)	1.0 (0.4 – 2.3)
**Formation or know some protocol of action** [b]	5.0 (3.0 – 8.1)*	0.8 (0.1 – 5.1)	0.9 (0.4 – 1.8)	1.3 (1.3 – 1.4)	0.9 (0.5 – 1.6)	0.2 (0.2 – 0.3)*

Values express the odds ratio and confidence interval of 95 % in parenthesis. Each column is an adjusted multiple logistic model, based on robust standard errors considering the 3 professionals groups as clusters.
CI: Correct Identification of FGM; DC: Detection of some case of FGM
[a] Data only available for 2001
[b] Data only available for 2004
* $p < 0.05$ on comparison with the reference category
